# The Gut Microbiota–Sex–Immunity Axis in Non-Communicable Diseases

**DOI:** 10.3390/life15101510

**Published:** 2025-09-25

**Authors:** Mario Caldarelli, Pierluigi Rio, Laura Franza, Sebastiano Cutrupi, Martina Menegolo, Francesco Franceschi, Antonio Gasbarrini, Giovanni Gambassi, Rossella Cianci

**Affiliations:** 1 Department of Translational Medicine and Surgery, Catholic University of Sacred Heart, 00168 Rome, Italy; mario.caldarelli01@icatt.it (M.C.); pierluigi.rio01@icatt.it (P.R.); sebastiano.cutrupi01@icatt.it (S.C.); martina.menegolo01@icatt.it (M.M.); antonio.gasbarrini@unicatt.it (A.G.); giovanni.gambassi@unicatt.it (G.G.);; 2 Fondazione Policlinico Universitario A. Gemelli, Istituto di Ricerca e Cura a Carattere Scientifico (IRCCS), 00168 Rome, Italy; 3 Emergency Department, Azienda Ospedaliero—Universitaria di Modena, Largo del Pozzo, 71, 41125 Modena, Italy; 4 Department of Emergency, Anesthesiological and Reanimation Sciences, Catholic University of Sacred Heart, 00168 Rome, Italy; francesco.franceschi@unicatt.it

**Keywords:** sex differences, gut microbiota, non-communicable diseases, cancer, autoimmunity, precision medicine

## Abstract

Non-communicable diseases (NCDs), including cancer and autoimmune, metabolic, cardiovascular, and neurodegenerative diseases, represent the leading cause of death globally and a growing healthcare burden. The gut microbiota (GM) has been recognized as a key biological component of host health that contributes to the maintenance of immune regulation, metabolic homeostasis, and epithelial barrier function. Several studies are now demonstrating that biological sex has an influence on both GM composition and function, which might explain sex differences in disease predisposition, course, and treatment response. Evidence from both clinical and experimental studies indicates that sex hormones, genetics, and lifestyle-related exposures interact with GM to influence the development and progression of most common NCDs. Some research suggests that estrogens promote diversity in GM with anti-inflammatory immune responses, while androgens and male-abundant taxa are associated with pro-inflammatory conditions. However, the evidence in humans is largely confounded by other variables (such as age, genetics, and lifestyle) and should be interpreted with caution. Unique GM metabolites, such as short-chain fatty acids and secondary bile acids, can have distinct, sex-specific effects on inflammation, metabolic regulation, and even antitumor immunity. While the existence of a sex–gut microbiota axis is gaining increased support, most studies in humans are cross-sectional epidemiological studies with limited mechanistic evidence and little consideration for sex as a biological variable. Future works should prioritize longitudinal, sex-stratified studies and utilize multi-omics integrated approaches to identify causal pathways. Ultimately, integrating sex differences into GM-based approaches could provide new avenues for personalized strategies for the prevention and treatment of NCDs.

## 1. Introduction

Approximately 70% of global deaths are due to chronic non-communicable diseases (NCDs), including cancer, autoimmune, metabolic, cardiovascular, and neurodegenerative [1].

Their prevalence is increasing worldwide, driven by aging, urbanization, and lifestyle changes, and they represent a substantial economic burden on healthcare systems [2].

In recent years, the gut microbiota (GM) has emerged as a central regulator of human health, shaping host metabolism, immune responses, and epithelial barrier function. Dysbiosis, i.e., the imbalance of composition and function of GM, has been implicated in the pathogenesis of NCDs [3,4,5]. Microbial products including short-chain fatty acids (SCFAs) and secondary bile acids, can modulate systemic inflammation, metabolism, and immune regulation and may serve as mediators between environmental exposures and disease development [6].

Emerging data suggest that biological sex is a key determinant of GM composition and function. Sex differences, hormonal changes, and sex-related lifestyle exposures contribute to the distinct microbial communities [7,8]. Considering the microbiota/immunity and sex hormones/microbiota relationship, a conceptual model named “microgenderome” was developed to indicate the interplay between sex hormones, the GM, and the immune system and to elucidate sexual dimorphic susceptibility to diseases [9]. Sex differences have been described for several NCDs, including a greater susceptibility to autoimmune diseases in women and an increased rate of metabolic and cardiovascular disorders in men [10,11].

In this narrative review, we summarize the current knowledge around the connection between sex–gut microbiota axis and NCDs.

We searched articles published between 2015 and 2025 in PubMed, Web of Science, and Scopus databases Using the keywords “sex differences,” “gut microbiota,” “non-communicable diseases,” and disease-specific terms (defined as “cancer”, “autoimmune”, “cardiovascular”, “metabolic”, “neurodegenerative”, “respiratory”, “kidney”, “liver”). Only English-language articles were included. We included both research articles and review papers, and emphasis was given to human studies and those in large animal models, though in vitro data were also included when applicable to gain some mechanistic insight. Screening of the reference lists of retrieved articles was also conducted to include further studies.

## 2. The Interplay Between Sex and Gut Microbiota Composition in Non-Communicable Diseases

The GM is a dynamic ecosystem, and its composition and activity is influenced by genetics, hormones, and environmental factors. The importance of biological sex in these determinants, provides a rationale for differences in microbial diversity, metabolic profiles, and host immune responses. Understanding these mechanisms is critical in explaining the different susceptibility to NCDs between men and women.

### 2.1. Genetics

Host genetics is a key driver of GM composition and function and interacts with sex to shape mucosal immunity. Data from animal and human studies suggest that genetic background not only limits microbial colonization but also shapes sex-related differences in microbiota profiles. Studies using murine models have provided insight into sex differences in GM. For example, Elderman et al. compared fecal microbiota and mucosal immune gene expression in two different mouse strains and discovered striking interactions between sex, genotype, and microbial composition. In female mice, there was a positive correlation between an increased amount of *Clostridium leptum* and an upregulation of genes responsible for leukocyte migration and inflammation responses. In contrast, in male mice there was an increase in *Faecalibacterium prausnitzii*, *Coprobacillus*, and *Clostridium ramosum*, which are taxonomic groups, linked to lymphocytes proliferation [8]. Kovacs et al. examined fecal samples from male and female mice across eight genetic lines and found that microbial composition was more strongly determined by genetic background than by sex. In other words, mice of the same genetic line were more similar to each other than to mice of the same sex [12]. Both studies demonstrate that microbial phenotypes showing sex differences are most often present in the context of specific genetic architectures.

It has been shown that certain polymorphisms in host genes can lead to enhanced microbial colonization and relative disease predisposition in a sex-dependent manner. A well-characterized example is the NOD2/CARD15 variants, which inhibit bacterial clearance promoting concurrent adherence-invasiveness of *E. coli* strains that are pathogen-associated with Crohn’s disease [12]. Since immune responses to pathobionts vary between sexes, these genetic susceptibilities may manifest differently in clinical presentation. Genome-wide association studies (GWAS) in humans have identified loci associated with GM composition by, along with some genes encoding pattern recognition receptors (PRRs), innate immune signaling mediators, and the major histocompatibility complex (MHC) [13]. This sex specific pattern in gene expression suggests, in combination with genetic polymorphisms, an important role in host-microbe interactions and sex-specific immune regulation.

A critical influence is also played by chromosomes. The X chromosome harbors several immune-related genes, including TLR7, FOXP3, and those encoding for many cytokines’ receptors. Because women carry two X chromosomes that are incompletely inactivated, they may have an increased dosage of certain immune-related genes, which in turn can affect microbial colonization and immune tolerance [14]. In contrast, Y chromosome haplotypes are associated with a variation in susceptibility to infection and autoimmunity, partly influenced by microbial interactions. For example, Y chromosome variants modulate the severity of disease through microbiota-dependent immune pathways in experimental models of autoimmune encephalomyelitis [15].

However, much more appears to be at stake in the interaction between genes and sex. Flak et al. introduced the term microgenderome, meaning that sex hormones shape the microbial community, while on the other hand, microbial enzymes and metabolites, such as β-glucuronidase, govern the bioavailability of systemic hormones [9]. Feedback is strongly driven by the host genotype. Yu et al., for instance, demonstrated that in postmenopausal women fecal β-glucuronidase activity correlates to urinary estrogen levels, suggesting that host–microbe–hormone interactions are modulated by endocrine changes and genetic determinants of microbial enzyme activity [16]. This bidirectional relationship complicates the interpretation of associations between microbes and hormones, as some observed effects may reflect microbial modulation of hormone levels rather than direct hormonal action. Studies have demonstrated that GM can influence gene expression in hosts through epigenetic mechanisms, including DNA methylation and histone modification. For example, butyrate a microbial metabolite functions as a histone deacetylase (HDAC) inhibitor, influencing the transcription of genes involved in inflammation and immune regulation. Butyrate has been shown to have stronger immunomodulatory effects in male than in female mice, indicating a possible sexually dimorphic pattern of epigenetic imprinting mediated by microbial products [17].

Polymorphisms of Toll-like receptors (TLRs) also affect immunity differently based on sex. Jabeen et al. showed that greater levels of phagocytic activity can be seen in female macrophages when compared to males, while differences in TLR4 mRNA expression might accentuate the underlying role of exposure in autoimmune diseases among women [18]. IL-6, a pleiotropic cytokine associated with chronic inflammatory response, is also modulated by the composition of GM. Estrogen and SCFAs appear to suppress IL-6 transcription, supporting the idea of a microbial–hormonal–cytokine axis that presents sex-dependent differences [19].

Fat mass and obesity-associated gene and peroxisome proliferator-activated receptor gamma (PPAR gamma) are examples of genes that affect metabolic regulation, involve the host genotype and interact with the GM. SCFAs promote the expression of PPAR gamma in white adipose tissue, with a differential effect in different sexes. These interactions may explain the differences between men and women in fat distribution and metabolic disease risk [20].

Unlike nuclear DNA, mitochondrial DNA (mtDNA) resembles bacterial genomes in its structure and is released during cellular stress, activating different innate immune responses. This is more obvious in men who have greater amounts of circulating mtDNA, making them more prone to chronic inflammatory diseases. Under the sex-specific influence, the GM modulates mitochondrial function and SCFA production, indirectly influencing systemic inflammation [21].

Here lies the complexity of host–microbe interaction mediated by genomic imprinting. Kagami et al. demonstrated that microbial signals could influence differentially methylated regions (DMRs) in imprinted genes such as IGF2 and MEG3, thereby affecting fetal development and immune regulation in a sex-dependent manner [22]. These findings suggest that the GM itself may contribute to epigenetic programming and disease predisposition, underscoring the importance of integrating both genetic background and sex-specific factors in microbiota research [22].

In summary, many interactions between host genetics and GM are strongly influenced by biological sex, shaping immune reactions, metabolic regulation, and determining disease susceptibility. From sex chromosomal differences and polymorphisms in immune-related genes to epigenetic modulation and mitochondrial signaling, sex-specific genetic profiles govern microbial colonization and function. This multilayered interaction highlights the necessity of conducting sex-stratified and genetic analyses in GM studies. Such approach will be crucial for the identification of specific microbial culprit and targeted interventions.

### 2.2. Sex Hormones

Most mechanistic data on hormone–microbiota interactions derive from rodent or in vitro models, while human studies are largely cross-sectional. Disruptions in this equilibrium, often occurring in the context of dysbiosis, may promote inflammatory and autoimmune diseases, typically occurring in a context of dysbiosis, may drive inflammatory and autoimmune diseases [23].

Research has revealed correlations between specific bacterial genera in the human gut and serum levels of sex hormones. For instance, elevated testosterone levels make the GM rich in *Ruminococcus*, *Acinetobacter*, and *Dorea*, while *Butyricimonas* and *Slackia* correlate with estradiol levels. Moreover, hormonal changes due to puberty, pregnancy, and menopause modify GM. Even hyperandrogenic conditions, such as polycystic ovary syndrome (PCOS), is linked to an increased amount of Actinobacteria and Proteobacteria and a loss of microbial richness [24]. As highlighted by D’Afflitto et al., the GM of healthy women has a predominance of Bacteroidetes, and a lower abundance of Firmicutes, compared to healthy men. High levels of estrogen and testosterone increase microbial diversity in women and men, respectively [25]. In addition, researchers associated progesterone with increases in *Bacteroides* and *Prevotella* spp. growth [26].

Collinearity stands as a primary methodological obstacle in the field. Indeed, along with sex, circulating hormone levels, and other covariates, including age, body mass index, diet, medication (such as antibiotics and exogenous hormones), and environmental factors all impact any association. giving rise to spurious associations. For example, both dietary patterns and BMI may produce varying features of circulating estrogens and gut microbiota structures, thus making it difficult to disentangle direct hormonal effects from potential confounding influences [4,27]. Such considerations will call for future research designs to stratify according to reproductive age and use of exogenous hormones, repeated endocrine measurements, and analytic strategies that address multicollinearity.

Most of the mechanistic information below comes from preclinical models (rodent and in vitro studies). Human data is increasingly available but is mostly cross-sectional, and caution is needed in the interpretation of the findings.

Sex hormones play a role in modulating intestinal barrier function. Specifically, estrogens can upregulate tight junction proteins, including occludin and claudin-1, which strengthen epithelial integrity and decrease microbial translocation [27]. In contrast, androgens may have neutral or damaging effects depending on the inflammatory context. In this respect, Van der Giessen et al. demonstrated that IBD models experienced increased barrier function following estradiol treatment, but not with androgens [27]. Similarly, Larauche et al. found that female mice exhibited sex-dependent differences in colon permeability; females were less tolerant to stress-induced barrier dysfunction [28].

The sex hormone–GM axis has been implicated in the sex-related differences in the incidence of colorectal cancer, which is much higher in men. Indeed, estrogen induces favorable changes in GM, enhancing the ratio of commensal bacteria to opportunistic pathogens and improving microbial diversity, thus promoting anti-tumor immunity. Instead, testosterone may increase the number of opportunistic pathogens and trigger inflammation, paving the way for tumor growth [29].

On the other hand, the GM itself has an impact on sex hormone metabolism through enzymes expressed by bacteria, such as β-glucuronidase [30]. Even in postmenopausal women, circulating estrogens depend on microbial β-glucuronidase activity, as suggested by the correlation between fecal β-glucuronidase activity with urinary estrogen levels [31]. Given the role of estrogen in the etiology of ovarian cancer, it has been hypothesized that the GM may take part in its development by increasing levels of active estrogen [32]. Choi and Choi described the relation between GM dysbiosis and ovarian cancer, due to the enhanced inflammatory response (e.g., IL-6 and Hedgehog pathways) and an increased risk of endometriosis induced by intestinal dysbiosis [33].

The interplay between GM and sex hormones seems to also plays a role in the pathogenesis of autoimmune diseases, such as type 1 diabetes (T1D). Mouse studies demonstrated that GM depletion may eliminate sex differences in glucose metabolism [34]. In particular, androgens seem to protect against T1D in a microbiome-dependent way and, as noticed by Marke et al., the transplant of GM from adult male mice into immature females increased testosterone levels, reducing the autoimmune response, as well as the morbidity due to T1D [24].

Furthermore, GM-derived metabolites exert a role in T1D pathogenesis. Sun et al. observed that SCFAs are reduced in non-obese diabetic (NOD) mice, compared to healthy controls. SCFAs modulate the release of cathelicidin-related antimicrobial peptide (CRAMP) by pancreatic beta cells. At this level, CRAMP contributes to immune homeostasis, by regulating pancreatic macrophages and generating Treg cells, thus protecting Non-Obese Diabetic mice against autoimmune diabetes. This effect was not observed in females [35].

Sex differences in SCFA-producing GM have been reported, with men exhibiting higher serum levels of SCFA than women and an increased amount of Tregs [23,36]. After an oligofructose-supplemented diet, significant changes in fecal community have been detected in female mice, with an increased abundance of Bacteroidetes. In males, the study revealed higher levels of fecal butyrate, hepatic IgA, and IL-6 after oligofructose consumption. These sex-specific responses to dietary interventions suggest that the impact of prebiotics on immunity may differ between males and females [37]. Gut dysbiosis may increase the production of pro-inflammatory mediators and reduce crucial neurotransmitters (e.g., serotonin, dopamine, and gamma-aminobutyric acid), causing neuroinflammation. In this context, sexual hormones show anti-inflammatory properties [38]. Furthermore, Wallis et al. noticed sex-specific interactions between the abundance of several GM genera within Firmicutes (e.g., *Clostridium*, *Lactobacillus*, and *Streptococcus*) and the clinical presentation of encephalomyelitis/chronic fatigue syndrome, a neuroimmune disorder [39].

Overall, sex hormones appear to provide a reasonable explanation for the sex differences seen in GM but current evidence does not support a causal role. Often, hormone measurements are made only at single time points and disregard temporal variations (i.e., menstrual cycle phases, pregnancy, and menopause or exogenous hormone usage). It would therefore be oversimplistic to put emphasis solely on hormones in this multifactorial relationship that must also include genetic, age-associated, diet-related, and environmental exposures [40,41]. Longitudinal studies stratified for sex with well-validated endocrine endpoints will be critical to assess the strength and directionality of the interactions between hormones and GM.

### 2.3. Exposome

In humans, sex differences in the GM may be the result of different exposure to extrinsic factors, such as foods, medications, and environmental pollutants, between men and women [42].

In a sex-specific analysis of 1135 gut metagenomes, Sinha et al. identified a larger number of antibiotic resistance genes in women, possibly due to a greater exposure to antibiotics, compared to men, who were in turn more likely to take cardiovascular medications. These findings are supported by experimental work in murine models showing genotype- and sex-dependent microbiota differences following antibiotic exposure [43]

As observed in animal studies, pollutants can modify the GM in a sex-specific manner [44]. For instance, the exposure to bisphenol A (BPA), an industrial chemical and endocrine-disruptor, increased Prevotellaceae in male mice, but not in females. BPA also promoted the enrichment of *Akkermansia* and *Methanobrevibacter* in the GM of male mice [45].

Diet affects the composition and metabolic profiles of GM in a sex-specific way. For example, high-fat diets (HFD), related to dysbiosis, chronic intestinal inflammation, and metabolic syndrome, impact on the levels of some taxa differently between the sexes. In female mice, HFD increased Lachnospiraceae species and decreased Rikenellaceae and *Ruthenibacterium lactatiformans*, whereas in males, a sex-specific overgrowth of Oscillospiraceae species, *Collinsella aerofaciens*, and *Ruthenbacterium* was observed [46]. Ruminococcaceae (e.g., *Ruthenibacterium*) play a role in the conversion of primary bile acids into secondary ones, such as deoxycholic acid, which drives malignant alterations in Lgr5-expressing cancer stem cells and may contribute to the adenoma-to-adenocarcinoma sequence [47].

Other relatively rare but potentially sex-specific modifiers of microbial communities may include smoking and alcohol consumption, whose effects, in particular on women, remain poorly characterized [48,49,50]. The gut dysbiosis caused by chronic cigarette smoking has been associated with systemic disorders, including gastrointestinal, cardiovascular, metabolic, and autoimmune ones [49]. Similarly, the GM disruption induced by alcohol consumption may drive pathological changes, contributing, for example, to the development and progression of alcohol-induced liver pathology [50].

Understanding how these environmental and hormonal influences intersect provides a foundation for developing sex-specific lifestyle and dietary interventions aimed at preserving gut health and reducing disease risk.

These sex-related differences in GM composition, diversity, and response to external factors are summarized in Table 1.

## 3. How the Microbiota Mediates the Effect of Sex on NCDs

The GM serves as a biological mediator through which sex differences influence the risk and progression of NCDs. These are predominantly mediated by microbial metabolites, like SCFAs, secondary bile acids, and trimethylamine-N-oxide (TMAO), that act modulating mucosal immunity and intestinal barrier integrity.

While human data are increasing, they are mostly cross-sectional, so caution is needed when interpreting their relevance. Indeed, most of the mechanistic information comes from preclinical models (rodent and in vitro studies).

Above and beyond metabolite production, GM exerts immunomodulatory effects through specific signaling pathways that interact with specific host receptors. SCFAs, butyrate, and propionate specifically bind to various G-protein-coupled receptors, such as GPR43 and GPR109A, leading to the proliferation of Tregs and secretion of anti-inflammatory mediators, such as IL-10 and TGF-β. Interestingly, the expression and responsiveness of these receptors seem to be sex-dependent. Docampo et al. found that GPR109A-deficient T cells promoted less graft-versus-host disease in male mice, suggesting that male SCFA-mediated tolerance may be more prominent [60]. Another study showed that SCFA stimulation enhanced Treg function to a greater degree in male immune cells [60]. This underscores the relevance of microbial signaling in establishing sex-oriented immune landscapes.

The effects of the GM on host physiology are not necessarily the same in both sexes. This is evident as microbial differences between the sexes can vary by chromosomal background and sex hormones, contributing to dimorphic outcomes in disease risk.

**Figure 1:**
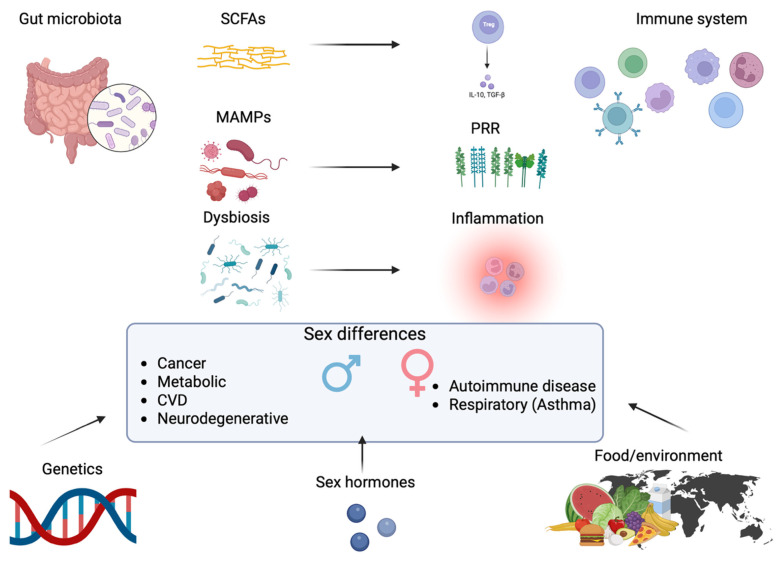
Interaction between GM, immune system, and sex in clinical outcomes of NCDs. Created with BioRender.com (https://app.biorender.com/illustrations/68a7466d65386958e3fb319d?slideId=dde30b8f-d88d-47ee-8700-b4f452828c7f, accessed on 29 August 2025).

Sex hormones also influence intestinal barrier integrity, which is modulated by GM. Estrogens generally enhance tight junction function, whereas androgens may impair barrier resilience in certain contexts. This interplay is summarized in Figure 1, which schematically illustrates the communication between the GM and the immune system, and how sex-specific factors (genetics, hormones, environment) influence disease outcomes.

**Figure 2:**
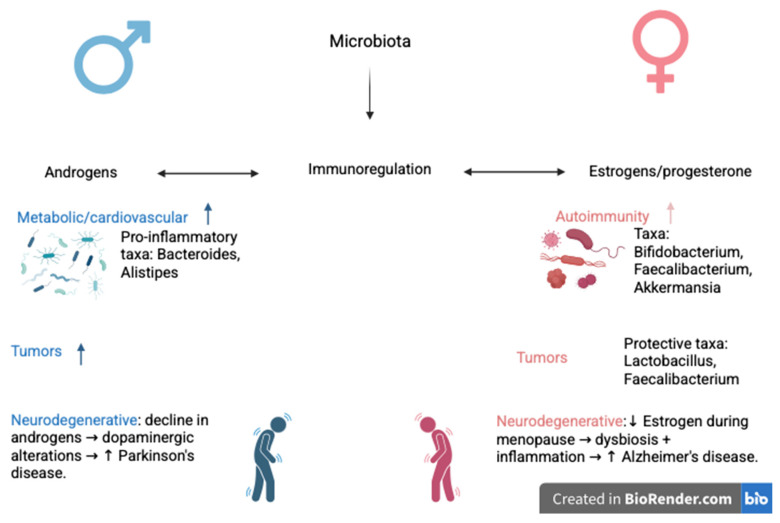
Sex-specific pathways through which microbiota composition and sex hormones (androgens in males, and estrogens/progesterone in females) influence immune regulation and disease susceptibility. For example, in males, the Bacteroides and *Alistipes* bacteria in the microbiota are associated with pro-inflammatory states, metabolic and cardiovascular effects, and an increased risk of Parkinson’s disease. For females, the estrogen/progesterone-associated microbiota, such as those found in *Bifidobacterium*, *Faecalibacterium*, and *Akkermansia*, are associated with autoimmunity, protective effects against tumors, and an increased risk of Alzheimer’s disease related to dysbiosis and inflammation after menopause. ↑ stands for “increase”; ↓ stands for “decrease”. Created with BioRender.com (https://app.biorender.com/illustrations/68ce5e5a0412ec45e879447e?slideId=d03490df-dcdc-4e6e-9b08-93c7e2adca2c, accessed on 20 September 2025).

To further illustrate the sex-specific impact of microbiota–hormone interactions on disease susceptibility, we developed a comparative schematic (Figure 2) summarizing key pathways and microbial taxa associated with immune regulation, neurodegeneration, and tumorigenesis in males and females.

GM communicates with the immune system mainly through metabolites (e.g., SCFAs) and microbial signals (MAMPs recognized by PRRs). Dysbiosis promotes chronic inflammation, and sex differences influence how these pathways affect disease susceptibility. Microbial fermentation of dietary fibers yields short-chain fatty acids (SCFAs), primarily acetate, propionate, and butyrate. These SCFAs, in turn, modulate host physiology by strengthening the intestinal epithelial barrier, stimulating regulatory T-cell responses, and downregulating the production of pro-inflammatory cytokines.

Experimental data indicate sex-dependent effects of SCFAs. In NOD mice, for instance, lower SCFA levels correlated with decreased pancreatic CRAMP expression and increased diabetes susceptibility in males but not in females. Human data on sex differences in circulating SCFAs are still scarce and heterogeneous (see Section 4 for disease-specific observations) [35].

Supporting evidence includes a report from Jakobsdottir et al. in which men had significantly higher fasting serum SCFA concentrations than women, corresponding to a higher number of regulatory T cells [36]. Altogether, these findings suggest that SCFA production and signaling represents an important axis of sex-specific immune regulation.

Secondary bile acids are another example of how microbial metabolism can interact with host sex to influence immunity. Secondary bile acids are produced because of the actions of bacterial bile salt hydrolases and influence mucosal immunity via IgA promotion and down-regulation of pro-inflammatory cytokines. Estrogens seem to amplify bile acid transformation and provide women with a unique metabolic and immunological framework. Ma et al., using murine experimental systems, reported that restoring GM to reverse sex-specific disruption of systemic factors in bile acid homeostasis, reduced aging associated inflammation, and suggested that estrogen mediated regulation may underlie women’s metabolic resilience [61].

Elevated plasma trimethylamine-N-oxide (TMAO) concentrations have been consistently associated with increased cardiovascular risk [62].

Microbial endocrinology is an emerging field in GM studies and refers to the ability of GM to synthesize, degrade, or modulate host neuroactive compounds and hormones. Certain bacterial taxa produce neurotransmitters such as serotonin, dopamine, and gamma-aminobutyric acid, which influence both central nervous system function and peripheral immune responses. Xu et al. mentioned that tryptophan metabolism via the microbiota-gut–brain axis is different in both sexes, with women manifesting lower serotonin availability and greater susceptibility to mood and metabolic disorders [63]. Barth et al. reported that sex hormones regulate neurotransmitter systems and influence brain architecture, hypothesizing that microbial neurochemical activity may contribute to sex-dependent disease phenotypes [64]. This strengthens the emerging view that endocrine signals derived from GM are essential for the sex–immunity–NCD axis.

Altogether these indicate that GM acts as a dynamic mediator of sex-specific immune and metabolic reactions. From receptor signaling and barrier modification to neuroendocrine crosstalk, microbial activity is inextricably linked with host physiology in a sex-dependent manner. Longitudinal studies like the Flemish Gut Flora Project and the American Gut Project have consistently shown sex-based differences in microbial diversity and metabolite profiles across populations [65,66,67]. This information highlights the need for sex-stratified clinical trials and mechanistic work to translate GM science into precision medicine strategies for NCDs.

To facilitate the interpretation of translational relevance, Table 2 summarizes the main findings on the sex–gut microbiota axis, distinguishing between evidence derived primarily from human cohorts from that based on preclinical studies.

## 4. Impact of the Sex–Gut Microbiota Axis on Specific Non-Communicable Diseases

The composition and functioning of the GM, in relation to biological sex, influences susceptibility, progression, and response to therapy for most common NCDs. The interaction between biological sex and the GM appears to be critically important especially in the case of cancer, autoimmune diseases, and metabolic and cardiovascular diseases [24,43].

### 4.1. Cancer

Cancer is one of the leading causes of mortality around the globe [68]. For several cancers, such as colorectal (CRC), pancreatic, and hormone-sensitive cancers, differences between sexes in incidence and prognosis, as well as response to therapy, have been well documented [80,81]. Besides genetic and hormonal factors, GM seems to act as a major modulator.

Women are endowed with estrogens that promote the growth of *Lactobacillus* and *Faecalibacterium* bacterial species recognized to produce anti-inflammatory metabolites and contribute to maintaining the integrity of the intestinal barrier [82]. These taxa lower predisposition to tumorigenesis, particularly in the case of CRC and pancreatic cancer [80]. In stark contrast, the presence of microorganisms enriched in *Bacteroides* and *Alistipes* n men has been associated with a pro-tumor environment; those are pro-inflammatory bacteria capable of increasing intestinal permeability [81].

Some microbial species are directly involved in neoplastic processes. *Fusobacterium nucleatum*, for instance, adheres to intestinal epithelial cells and activates the Wnt/β-catenin pathway via the FadA adhesin, thereby stimulating cell proliferation [83]; in addition, the Fap2 protein interacts with the TIGIT receptor on T and Natural killer cells, inhibiting antitumor immunity [84]. Enterotoxigenic *Bacteroides fragilis* induces pro-proliferative long non-coding RNAs (*Bacteroides fragilis*-associated lncRNA1) by activating the RHEB/mTOR pathway [85]. Colibactin-producing *Escherichia coli* can damage DNA and induce genomic instability that promotes neoplastic transformation [86].

Metabolites from GM can also shape the tumor microenvironment. Deoxycholic acid (DCA), a secondary bile acid produced by *Clostridium scindens*, can hinder the functions of tumor-infiltrating CD8^+^ T cells by inhibiting effector cytokine (IFN-γ, TNF-α) production and by activating the IL-6/STAT3 pathway for promoting metastasis [87,88,89,90]. In contrast, indole-3-propionic acid obtained from tryptophan fermentation by *Lactobacillus johnsonii* and *Clostridium sporogenes* has been suggested to enhance antitumor immune responses and to boost the efficacy of immunotherapy [71].

### 4.2. Autoimmune Disorders

The predominant mechanism by which GM dysbiosis contributes to the development and progression of autoimmune diseases is the disruption of immune pathways [91]. Probiotic imbalances impair immune homeostasis by upregulating pro-inflammatory cytokines (IL-1β, 6, TNF-α, IL-17, IL-12, IFN-γ) and downregulating anti-inflammatory cytokines (IL-10, IL-4, IL-13, TGF-β, IL-1ra). This has been clearly documented among patients with systemic lupus erythematosus (SLE) [92].

In SLE, the activation of TLRs on dendritic cells and macrophages results in the release of cytokines, such as IFN-α, IFN-β, IL-6, and IL-23, which play a significant role in the development and progression of the disease [93]. However, supplementation with *Bifidobacterium bifidum* has been shown to balance out the Treg/Th17/Th1 ratio and prevent the over-activation of CD4^+^ lymphocytes [94]. Moreover, dysbiosis has been implicated in systemic autoimmunity as well as in organ-specific disease. For instance, in patients with SLE, *Ruminococcus gnavus* has been found to increase substantially with higher serum levels of antibodies targeting its antigens. This microbial signature has been specifically linked to lupus nephritis, in support of the idea that perturbations of the GM could impact renal disease via increased immune activation and through mimicking host molecular structures [95]. Most of the mechanistic insights into SLE and GM come from lupus-prone mouse models, while human studies are fewer and report heterogeneous results. These findings reinforce the concept that microbial mimicry may be a key driver of sex-skewed autoimmunity, particularly in diseases such as SLE where microbial antigens selectively interact with the female immune profile. The data also supports the general notion that dysbiosis may have an impact not only on the peripheral but also on target organ responses in autoimmune diseases [96]. One of the ways in which dysbiosis favors autoimmunity is through molecular mimicry, in which microbial epitopes are structurally similar to host antigens and can stimulate immune cross-reactivity [69]. For example, *Bacteroides fragilis*, *Prevotella copri*, *Candida albicans*, and *Streptococcus sanguis* peptides have been reported to mimic collagen and synovial proteins [97].

Hansen et al. found an increasing number of microbial peptides that mimic host self-antigens, further supporting molecular mimicry, a hallmark of dysbiosis, as a pathogenic factor in autoimmunity [98].

For instance, peptides derived from GM species, such as *Akkermansia muciniphila* and *Ruminococcus gnavus* have been shown to mimic pancreatic beta-cell antigens, thereby establishing a link between these bacteria and the development of type 1 diabetes [99].

Moreover, the finding that citrullinated fibrinogen peptides found in the synovial tissue of rheumatoid arthritis (RA) patients are very similar to bacterial antigens and that demyelination is driven by molecular mimicry between myelin and certain types of microflora further emphasizes the role of microbe-driven autoimmune responses in the development of autoimmune diseases [62]. Yet, while most clinical data come from human cohorts, mechanistic causality is still largely inferred from animal studies.

### 4.3. Metabolic and Cardiovascular Diseases

Metabolic and cardiovascular diseases (CVDs) represent a major global health burden. Evidence suggests sex-based differences in GM composition and activity may influence their onset and progression. The gut–sex–immunity axis may help explain sex differences in metabolic syndrome, obesity, type 2 diabetes mellitus (T2DM), and cardiovascular risk [24,43,70]. The supporting literature in this field is based on several human cohorts and longitudinal studies, making it more translationally relevant. However, some mechanistic insights continue to stem from preclinical experiments.

Obese women have increased levels of Firmicutes and decreased levels of Bacteroidetes, while in obese men, their GM is enriched with *Prevotella* and Proteobacteria [72,100]. Women tend to accumulate more subcutaneous adipose tissue, enjoying relative protection against cardiovascular risk, while male accumulation of visceral adiposity and related systemic inflammation produce greater metabolic and cardiovascular risk [101]. Furthermore, sex hormones seem to influence these associations. Estrogens improve insulin sensitivity and alter bile acid metabolism, while androgens, especially when in excess, can promote dysbiosis and insulin resistance [73].

The GM has a significant effect on lipid profiles and cholesterol metabolism, as it changes the composition of bile acids and produces SCFAs. While butyrate and propionate enhance insulin sensitivity, secondary bile acids such as deoxycholic acid may exacerbate inflammation and endothelial dysfunction [102]. Other sex differences are also obvious: pre-menopausal women, for instance, have SCFA-producing taxa that favor a better lipid profile compared to men, hence lower cardiovascular risk [103].

Epidemiological studies link higher plasma TMAO to endothelial dysfunction, platelet hyperreactivity, and increased risk for myocardial infarction and stroke. For example, in the Multi-Ethnic Study of Atherosclerosis, baseline TMAO predicted incident atherosclerotic events over 11 years [62]. Furthermore, an interventional study conducted at the Cleveland Clinic found that a diet rich in red meat raised plasma TMAO threefold and reduced its renal clearance [104]. Notably, women generally show lower TMAO levels than men, a difference that might be due to sex-specific gut microbial composition and estrogen-based modulation of hepatic flavin monooxygenase activity [74].

Dysfunctional fat tissue refers to low-grade systemic inflammation, one of the causes of obesity-related insulin resistance and cardiovascular risk [105]. Another modulator is gut microbiota dysbiosis, which causes increased intestinal permeability and induces metabolic endotoxemia, which in turn augments inflammation in adipose tissue and systemic immune activation [106,107]. Overall improved intestinal permeability, as well as an increased translocation of lipopolysaccharide (LPS), stimulates repetitive activation of the general immune system. These conditions are likely more protective in women, while in men, a greater abundance of pro-inflammatory taxa (e.g., *Bacteroides*, *Alistipes*) exacerbate the cardiovascular inflammation [108].

Sex-specific GM signatures and their metabolites may represent biomarkers for metabolic and cardiovascular risk stratification. Probiotic and prebiotic interventions targeting SCFA production or TMAO reduction have shown promise in experimental models [109], but sex-specific clinical trials are still lacking. Incorporating sex as a biological variable in GM-based interventions could optimize strategies for the prevention and treatment of obesity, T2DM, and CVD.

## 5. Neurodegenerative Diseases

The gut–brain axis encompasses a bidirectional communication network among the GM, the immune system, and the brain; that influences neuroinflammation, neuronal health, and behavior. Sex hormones and sex chromosome effects alter GM composition and metabolite production, which then impact blood–brain barrier function, microglial activity, and neuroimmune responses. Considering sex as a biological factor is thus critical when examining GM–brain relationships and when developing GM-based therapies [38,110]. GM affects neurodegenerative diseases, and most mechanistic insights surrounding this notion come from rodent models. Studies in the human GM in terms of Alzheimer’s and Parkinson’s diseases are beginning to emerge, but these studies are still insufficient and not stratified by sex.

Both diet and nutrition represent other factors that exert a considerable influence over gut–brain interactions. The eating pattern with the highest positive association with microbial diversity and with the highest concentration of bacteria that produce short-chain fatty acids, probably endowed with neuroprotective and anti-inflammatory properties, is the Mediterranean diet and high fiber [111,112]. Conversely, Western-style diets containing abundant saturated fats and refined sugars have been related to gut dysbiosis and increased permeability and, thus, systemic inflammation, worsening the neurodegenerative processes [113].

It is important to note that diet can independently influence gut microbiota and may overlap with microbiota alterations reported in AD, PD, and MS.

### 5.1. Alzheimer’s Disease

Alzheimer’s disease (AD) is the primary cause of neurodegeneration in humans and exhibits a distinct sexual bias, with women experiencing higher incidence and faster cognitive decline than men [75]. The difference in AD is influenced by a variety of factors, such as hormonal changes, sex chromosomes, and differences in immune responses. The gut–brain axis has been implicated in the risk of developing AD and its progression.

Estrogen is a neuroprotective agent that exerts its function through antioxidation, modulation of synaptic plasticity, and a decrease in neuroinflammation [114]. The sudden estrogen fall during menopause is correlated to the production of amyloid-β (Aβ) aggregates and tau hyperphosphorylation, which in turn leads to women’s increased vulnerability to AD. On the other hand, androgens in men are connected with the control of microglial activation as well as neuronal survival, but the decrease in testosterone levels with aging has also been linked to increased AD risk [115].

Dysbiosis has been reported in AD patients, with reduced levels of beneficial taxa such as *Faecalibacterium* and *Bifidobacterium*, and increased taxa with pro-inflammatory properties such as *Escherichia/Shigella* [116]. These changes are associated with decreased production of SCFAs and increased translocation of bacterial endotoxins, both of which evoke systemic inflammation and neuroinflammation. GM-derived metabolites also influence tryptophan metabolism, which impacts the neurotoxic kynurenine pathway products, a process that appears to differ by sex [117].

A growing body of evidence suggests that GM modulates AD in sex-dependent manner. Women appear more susceptible to pro-inflammatory signaling from GM, in part due to increased gut permeability and cytokines once estrogen is withdrawn from the system [118]. In murine models, females with AD-like pathology show greater microglial activation and heightened responses to microbial metabolites than males [76].

Thus, diet and nutritional interventions may represent promising strategies to modulate gut microbiota and potentially influence neurodegenerative processes.

### 5.2. Parkinson’s Disease

Parkinson’s disease (PD) is the second most prevalent neurodegenerative condition and like Alzheimer’s disease, exhibits significant sex differences in prevalence, clinical characteristics, and disease progression. PD is diagnosed in men 1.5 times more often than in women, and women tend to present with less severe motor signs but a greater burden of nonmotor symptoms such as depression, anxiety, and sleep disorders [119]. The sex differences in clinical presentation of PD are proposed to emerge from the interaction between sex hormones, immune system, and genetics, all of which have been shown to correlate with GM composition and function. 

Estrogens exert neuroprotective effects by altering mitochondrial functions, decreasing oxidative stress, and reducing neuroinflammation. Estrogens also promote the survival of dopaminergic neurons, potentially explaining the delayed development of PD and slower progressive decline in pre-menopausal women [120]. After menopause, the decline in estrogens stimulates the neurodegenerative processes; however, similarly, androgens and their decline in men become a risk factor for dopaminergic loss and motor symptoms [121].

There is growing evidence that gut dysbiosis is an early marker of PD, with changes evident years before motor symptoms arise. Patients with PD generally have reduced abundance of SCFA-producing microbes *Faecalibacterium prausnitzii* and *Roseburia*, and an enrichment of pro-inflammatory taxa such as *Enterobacteriaceae* and *Akkermansia* [122,123]. These changes lead to increased permeability in the gut, systemic inflammation, and enhanced misfolding of α-synuclein in the enteric nervous system, an effect which may project to the CNS via the vagus nerve.

### 5.3. Multiple Sclerosis and Other Demyelinating Diseases

Multiple sclerosis (MS) is a chronic inflammatory disease-causing demyelination of the central nervous system, one that has considerable sex bias. Women are two to three times more likely than men to be diagnosed with MS; however, men often have a more aggressive course of disease and faster progression to disability [77]. Similarly, other demyelinating diseases such as neuromyelitis optica spectrum disorder (NMOSD) show sex-associated differences regarding prevalence and severity. The interaction among sex hormones, genetic susceptibility, immune modification, and GM is increasingly recognized as a key aspect that impacts disease susceptibility and clinical heterogeneity.

Estrogens enhance immune tolerance by shifting T cell polarization towards regulatory phenotypes and reducing Th1/Th17-mediated inflammation. Therefore, relative immune protection exists for women during pregnancy and premenopausal states [124]. The abrupt decrease in estrogens arising from menopause has been linked to increased number of relapses and increased rate of disease progression. Androgens, although less studied in this context, appear to have anti-inflammatory effects and support remyelination; furthermore, low testosterone levels in men with MS have been related to higher disease activity [125].

Several studies have found that patients with MS exhibit dramatic GM dysbiosis compared to healthy controls, with diminished abundances of beneficial taxa like *Faecalibacterium*, *Bifidobacterium*, and *Prevotella*, and increased abundances of pro-inflammatory genera like *Akkermansia* and *Methanobrevibacter* [126,127]. Dysbiosis is associated with impaired production of SCFAs, increased intestinal permeability, and activation of systemic immune pathways that promote CNS inflammation and demyelination.

The GM is sexually dimorphic in shaping immune function at several levels. For example, in experimental autoimmune encephalomyelitis (EAE), a mouse model of MS, there is greater expansion of pro-inflammatory taxa and enhanced Th17 responses in females compared to males, which also leads to higher disease incidence [128].

The GM of male mice is protected from EAE through androgen-mediated microbial changes, indicating that sex hormones can shape the microbiota in ways that reduce susceptibility to disease.

Collectively, these findings indicate that sex and GM influence susceptibility, clinical course, and response to treatment in major neurodegenerative diseases. Sex hormones and chromosomal features interact with GM composition and function. This intersection influences neuroinflammation, blood–brain barrier permeability, and neuronal survival. Dysregulation of microbial metabolites, such as SCFA, bile acids, and tryptophan derivatives, may differentially affect men and women.

In summary, evidence connecting GM to neurodegeneration remains predominantly preclinical. Large, longitudinal, sex-stratified human studies are needed to validate these findings and to assess their translational relevance.

## 6. Other Non-Communicable Diseases

The GM and lung axis is influenced by sex. Asthmatic adult women show increased Th2 immune response and reduced microbial diversity [79,129]. The opposite holds true in men with COPD, who show increased Proteobacteria and smoking-related dysbiosis, which highly correlates with systemic and airway inflammation as well as severity of disease [79]. Experimental allergen-induced models demonstrate sex-specific changes in gut and lung microbiome (e.g., Firmicutes, Bacteroidetes, Proteobacteria), implying a role of sex hormones in microbial ecology and airway immune tone [129].

Observational studies have linked gut microbiota-derived metabolites like indoxyl sulfate and p-cresyl sulfate to CKD progression, with experimental evidence indicating levels may vary by sex [130]. Studies on animal models show that microbiota regulates renal gene expression in a sex-specific manner: in males lacking microbiota (germ-free), colonization with intestinal microbes leads to an increase in Verrucomicrobia, while in females there is an enrichment of *Akkermansia muciniphila*, which exerts a protective effect against renal damage [131]. Direct causation and sex-specific regulation remain unproven in humans and require further investigation.

Metabolic Associated Fatty Liver Disease (MAFLD) and Metabolic Dysfunction-Associated SteatoHepatitis (MASH) have specific sex-associated relationships with GM. Premenopausal women are protected by estrogen levels, which enhance SCFA producers and decreases intestinal permeability, which is lost after menopause. Large population studies have reported sex differences in GM composition in patients with MAFLD. Specifically, there appears to be a decreased microbial diversity and *Streptococcus*, *Bifidobacterium*, and *Dialister*, as well as *Phascolarctobacterium* abundance in men, while other Lachnospiraceae and Ruminococcaceae in women are more common [78]. These results indicate sex-specific microbial risk profiles and potential targets for personalized approaches.

## 7. Conclusions and Future Perspectives

Mounting evidence indicates that GM exerts an important role as mediator of the documented sex-based differences in susceptibility, progression, and treatment outcomes for the most common NCDs. Microbial composition and function are influenced by sex chromosomes, hormones, and lifestyle exposures. The specific role of hormones remains unclear. Most human evidence is associative and can be affected by confounding factors such as age, dietary habits, and medication use. Emphasizing the role of hormones too much may oversimplify these complex interactions. Careful, longitudinal studies with sex-based stratification are needed to clarify the underlying mechanisms.

Research in the future should pursue three main directions. First, well-designed clinical studies that are sex-stratified are needed to validate microbial biomarkers and therapeutic targets for personalized approaches. Second, integrative approaches, such as metagenomics, metabolomics, and immuno-profiling or multi-omics, will be invaluable to delineate causal pathways linking sex, GM, and disease outcomes [132]. Lastly, new interventions such as precision probiotics, postbiotics, dietary interventions, and fecal microbiota transplants need to be designed and studied in a sex-specific way to be able to maximize efficacy while minimizing harm [67].

A new and exciting discipline tries to develop probiotics and postbiotics for sex-specific precision microbiome-based approaches. Recent clinical evidence suggests that probiotic supplementation may have sex-dependent immunomodulatory effects. For example, in a double-blind, randomized, placebo-controlled trial involving community-dwelling older adults, probiotics induced significant modulation of gut taxa and peripheral immune cell subsets, affecting monocytes, dendritic cells, and CD4^+^ T cells [133] in a sex-specific manner. In addition, in women diagnosed with polycystic ovary syndrome, a beneficial effect on the hormonal profile, SHBG levels, and BMI was observed after a 12-week intervention with a multi-strain probiotic, further confirming the importance of sex-relevant microbiome modulation [134]. Moving beyond probiotics, postbiotics are now gaining recognition for their clinical potential, and recent reviews have highlighted their capacity to regulate immune and metabolic pathways [135]. Furthermore, mechanistic studies indicate that probiotic-induced modulation of ovarian hormone metabolism [136] and microbial enzyme activities such as β-glucuronidase [137] could be harnessed to design targeted interventions. Additionally, experimental data show that probiotic treatment after traumatic brain injury induced sex-specific neuroprotection and altered short-chain fatty acid production, underscoring the importance of sex as a determinant of microbial metabolite activity [138]. Altogether, these findings strengthen the rationale for the development of sex-stratified probiotic and postbiotic formulations that may enhance efficacy while minimizing adverse effects in the prevention and treatment of NCDs.

Males exempted via a multi-omics approach to the definition of sex, plus stratification by menopause status, must be considered among the most critical areas in the future. There are marked differences in the gut microbiota composition, microbial metabolite profiles, and immune responses between pre- and postmenopausal women, caused by the very rapid decline of circulating estrogens [139]. Menopause is associated with lower microbial diversity and altered bile acid metabolism, as well as a distinct short-chain fatty acid signature compared to premenopausal states, as indicated by recent metabolomics and metagenomics studies [140].

In conclusion, different microbiota profiles are associated with sex, but sex does not determine microbiota composition directly, and the role of the sex chromosomes should not be overrated. Microbial diversity results from various interplays between genetic material, hormones, dietary habits, and life preferences. However, viewing sex as a biological variable could open some new possibilities for personalized prevention and management of NCDs across the bench-to-bedside divide in the age of precision medicine.

## Figures and Tables

**Table 1:** This table summarizes representative findings on how sex hormones and chromosomal differences may influence GM composition, diversity, and responsiveness to variables such as diet and hormonal cycles. The data are drawn from a mix of human studies, animal models (e.g., mice, pigs), and in vitro experiments. As such, the results should be interpreted with caution, especially when extrapolating to human physiology. References include both primary research articles and review papers to provide a broader perspective.

Aspect	Typically Observed in Females	Typically Observed in Males	References
Hormonal Influence	Higher estrogen and progesterone levels; cyclical variation (menstrual, pregnancy, menopause)	Higher testosterone and androgens; more stable hormonal profile post-puberty	[51,52,53,54]
Dominant Bacterial Taxa	↑ *Bifidobacterium*, ↑ *Faecalibacterium*, ↑ *Akkermansia*, ↑ *Lactobacillus* (during pregnancy), ↑ *Parasutella* (mice) *	↑ Bacteroides, ↑ *Alistipes*, ↑ *Clostridium*, ↑ *Prevotella*, ↓ *Turicibacter* (mice) *	[24,55] *
Taxa Associated with Estrogen	↑ Clostridia, ↑ Ruminococcaceae (express β-glucuronidase, reactivating estrogens)	Variable; higher estrogen activation post-menopause due to GM composition	[31,56]
Taxa Associated with Androgens	↓ *Akkermansia* and ↓ Ruminococcaceae (pigs) *; ↑ *Streptococcus*, *Escherichia/Shigella* (mice) *	May promote more stable GM at moderate levels (mice) *	[57,58] *
GM Diversity	Generally higher, especially pre-menopause; fluctuates with hormonal phases	Slightly lower overall, but more stable over time	[24,59]
Lifestyle Sensitivity	More sensitive to micronutrient deficiencies, restrictive diets, hormonal contraception (in vitro) *	More influenced by high-fat diets, smoking, alcohol consumption (in vitro) *	[46,49,50] *

* findings based on animal models (e.g., mice, pigs), in vitro experiments, or review articles. Interpretations should be made cautiously when extrapolating to humans. ↑ stands for “increase”; ↓ stands for “decrease”.

**Table 2:** Summary of the main findings on the sex–gut microbiota axis, differentiating between findings primarily from human studies and those derived from preclinical (animal or in vitro) models. The table presents differences in microbiota composition, hormone–microbiota interactions, and disease-specific mechanisms (i.e., autoimmune, cancerous, metabolic, cardiovascular, and neurodegenerative diseases), thereby advocating for sex-stratified approaches in translational research. ↑ stands for “increase”; ↓ stands for “decrease”.

Aspect/Disease Area	Findings Mainly from Human Studies	Findings Mainly from Preclinical Studies (Animal or In Vitro)	Key References (from Text)
Gut microbiota composition and sex differences	Population cohorts (Flemish Gut Flora Project, American Gut Project) show sex-based diversity: women ↑ diversity, men ↑ *Bacteroides*, *Alistipes*	Murine models: strain- and sex-dependent differences (e.g., C57BL/6 vs. BALB/c); microbial phenotypes shaped by sex–genotype interactions	[8,16,68]
Sex hormones and microbiota	Correlation between estrogens/testosterone and taxa (*Butyricimonas*, *Slackia*, *Ruminococcus*); human data in puberty, pregnancy, menopause, PCOS	Mechanistic: β-glucuronidase regulates estrogen levels; estradiol protective in IBD; androgen–GM link in T1D mouse models	[24,25,28,29]
Autoimmune diseases (SLE, T1D, RA)	SLE: dysbiosis with *Ruminococcus gnavus* expansion linked to nephritis; RA: microbial mimicry with citrullinated peptides; limited stratified cohorts	NOD mice: SCFA depletion promotes T1D; GM transfer from males protects females; EAE: female mice more susceptible via GM shifts	[69,70]
Cancer	CRC and pancreatic cancer: women enriched in protective taxa (*Lactobacillus*, *Faecalibacterium*); men enriched in pro-inflammatory *Bacteroides*, *Alistipes*	Mechanistic: *Fusobacterium nucleatum* (FadA, Fap2), *B. fragilis* toxins, colibactin+ *E. coli*; bile acids and indole metabolites modulate tumor immunity	[71]
Metabolic and cardiovascular diseases	Human cohorts: obese women ↑ Firmicutes, obese men ↑ *Prevotella*/Proteobacteria; women generally lower TMAO levels; estrogens improve insulin sensitivity	Murine studies: HFD induces sex-specific taxa shifts; microbial metabolites (SCFAs, bile acids) interact with estrogen/androgen signaling	[72,73,74]
Neurodegenerative diseases (AD, PD, MS)	AD: dysbiosis (↓ *Faecalibacterium*, ↑ *Escherichia/Shigella*); PD: altered taxa precede symptoms; MS: limited sex-stratified cohorts	Rodent models: estrogen neuroprotection via GM; sex-dependent SCFA/tryptophan metabolism; EAE: androgen-protected male microbiota	[75,76,77]
Other NCDs (respiratory, kidney, liver)	Asthma (women: Th2 ↑, diversity ↓); COPD (men: ↑ Proteobacteria, smoking-induced dysbiosis); MAFLD: sex-specific taxa (*Streptococcus*, *Dialister*, *Ruminococcaceae*)	CKD/MAFLD models: male vs. female colonization with Akkermansia vs. Verrucomicrobia; BPA/pollutants induce sex-specific dysbiosis	[78,79]

## Data Availability

No new data were created or analyzed in this study.

## Abbreviations

AD	Alzheimer’s disease
BPA	Bisphenol A
CKD	Chronic kidney disease
CRAMP	Cathelicidin-related antimicrobial peptide
CVD	Cardiovascular disease
EAE	Experimental autoimmune encephalomyelitis
GM	Gut microbiota
GWAS	Genome-wide association studies
HDAC	Histone deacetylase
HFD	High-fat diet
LPS	Lipopolysaccharide
MAFLD	Metabolic Associated Fatty Liver Disease
MAMPs	Microbe-associated molecular patterns
MASH	Metabolic dysfunction-Associated SteatoHepatitis
MHC	Major histocompatibility complex
MS	Multiple sclerosis
NCD	Non-communicable disease
NOD	Non-obese diabetic (mouse model)
NMOSD	Neuromyelitis optica spectrum disorder
PCOS	Polycystic ovary syndrome
PD	Parkinson’s disease
PPARγ	Peroxisome proliferator-activated receptor gamma
PRRs	Pattern recognition receptors
SCFA	Short-chain fatty acids
SLE	Systemic lupus erythematosus
T1D	Type 1 diabetes
T2DM	Type 2 diabetes mellitus
TMAO	Trimethylamine N-oxide
TLR	Toll-like receptor
Treg	Regulatory T cells
